# Human sounds and associated tonality disrupting perceived soundscapes in protected natural areas

**DOI:** 10.1038/s41598-025-08524-y

**Published:** 2025-08-06

**Authors:** Tin Oberman, Arianna Latini, Francesco Aletta, Giacomo Gozzi, Jian Kang, Simone Torresin

**Affiliations:** 1 Institute for Environmental Design and Engineering, University College London, 14 Upper Woburn Place, London, WC1H 0NN UK; 2 Department of Construction, Civil Engineering and Architecture (DICEA), Università Politecnica delle Marche, Ancona, IT Italy; 3 Silenzi in Quota, Trento, IT Italy; 4 Department of Civil Environmental and Mechanical Engineering, University of Trento, Via Mesiano 77, 38123 Trento, IT Italy

**Keywords:** Soundscape, Protected natural area, Soundwalk, Overtourism, Psychoacoustics, ISO 12913, Anthropogenic noise, Psychology, Environmental social sciences, Engineering

## Abstract

In protected natural areas (PNAs), at popular scenic spots, visitors often contribute to noise pollution through their behaviour. The decibel-focused monitoring doesn’t capture the quality of an acoustic environment, human behaviour and perception. A mixed-methods framework, based on the ISO 12913 series, was tested in four European PNAs to address this gap. During five soundwalks (7–12 km long) organised by the *Silenzi in Quota* initiative, 443 questionnaires were gathered across 28 evaluation points, alongside corresponding binaural measurements. Acoustic environments as silent as L_Aeq_ = 31 dB and as loud as L_Aeq_ = 76 dB were observed, eliciting perceptions from very calm to chaotic. Psychophysical measures (loudness, sharpness, roughness, fluctuation strength and tonality) were calculated. The impact of the perceived sound source dominance, visual landscape quality and psychophysical and environmental acoustic features on the perceived soundscape pleasantness and eventfulness was analysed via Linear Mixed-Effects Models (LMMs). Perceived sound source type data- and psychophysical data-based models demonstrated higher predictive power than those based on sound pressure level metrics. Amongst the sounds of nature, water sounds demonstrated the strongest association with higher pleasantness and eventfulness. Unlike in urban context, presence of human sounds, associated with increased tonality, was the major factor driving the perception of chaotic soundscapes, revealing the detrimental effect of human behaviour on the experience of PNAs.

**Supplementary Information:**

The online version contains supplementary material available at 10.1038/s41598-025-08524-y.

## Introduction

Anthropogenic noise is a major source of pollution affecting urban and natural landscapes around the world, recognised as an emerging issue of environmental concern by the United Nations Environment Programme (UNEP) in 2022^1^. While this issue is typically associated with urban areas, Protected Natural Areas (PNAs) are disrupted by noise as well^2^ with spots of outstanding natural beauty being their most fragile parts when they get exploited as tourist attractions, often resulting in degraded biodiversity^3^. Beyond preserving endangered landscape and enabling biodiversity conservation, PNAs are an essential resource allowing visitors to experience positive well-being effects of being in nature^4–6^ which makes them of prime interest for soundscape research, as understood in ISO 12913^7–9^ Acoustics - Soundscape series.

Part 1 of the ISO 12913 series defines soundscape as an acoustic environment, as perceived by the people in context^7^. This has, in a way, set up soundscape research as a human perception-focused, mixed methods-based discipline, developed around questionnaire tools and/or interviews and environmental acoustics measurements, investigating how people perceive sounds of a place. However, research and policy in PNAs have traditionally approached sound-related human activity primarily from a noise mitigation perspective, mostly focusing on traffic-related noise reduction and mainly relying on physical metrics such as sound pressure levels. However, this approach overlooks a critical dimension: how humans perceive and respond to sound in context. Growing evidence suggests that natural sounds can enhance well-being^10–15^ and that perceptual outcomes from noise depend not only on energy-related metrics but also on context and meaning. Despite this, perceptual and experiential aspects of the acoustic environment remain underexplored in natural settings, especially in comparison to the extensive body of urban soundscape research - highlighting a notable research gap.

A holistic investigation of environmental sounds is characteristic of the soundscape approach outlined in the ISO 12913, which was implemented in this study by conducting participative socio-acoustic surveys and binaural acoustic measurements to characterise an acoustic environment in PNAs and observing its effect on human perception. In the two subsections below, we first briefly examine how soundscape issues in PNAs are addressed in policy and research, followed by introducing the ISO 12913 soundscape framework, which provides the conceptual and methodological foundation for this study’s pioneering application in mountainous protected areas.

### Acoustic quality in PNAs: positive soundscape in policy and research

The international institutions such as the United Nations Educational, Scientific and Cultural Organisation (UNESCO) and the International Union for Conservation of Nature (IUCN) have developed protection guidelines to be applied to valuable natural areas around the world, requiring management strategies and often sharing the risk of overtourism^16–19^. The associated management plans, usually built on historical field data on the physical characteristics of an area and social/cultural/economic significance, include aspects related to aesthetics and visitors’ experience^20^. Regarding the appraisal of positive sound sources in the management documents, natural sounds and noise occasionally get mentioned but those mentions usually provide little or no actionable points. This issue will be briefly illustrated later in this study in the description of the case study sites (see Methods). This implies that more research is needed to characterise the acoustic environments and soundscapes of PNAs so they could be implemented in the protection documentation in a meaningful way, informing strategies to manage visitors’ behaviour and the risks of overtourism.

Within the European Noise Directive published in 2002^21^ and the subsequent European Environment Agency Technical report No 4/2014 Good Practice on Quiet Areas^22^ PNAs are treated together with other exurban areas, sharing criteria for categorisation as quiet areas and the associated ‘quiet targets’, where soundscape is one of the key perceptual indicators alongside the environmental acoustic measurements. It is important to note that, in general, exurban areas receive less attention than urban ones and, while acknowledged as very important, soundscape criteria are mentioned in a very vague manner. This is due to the a lack of comparable perceptual data between the studies as many different approaches were observed to characterize the soundscape construct, such as tranquillity and wildness^23,24^ or the perceived affective quality^25^.

Studies investigating environmental sounds in PNAs are often focused on reporting sound pressure level-derived metrics^26–29^ and sound source type characterization as the main qualitative feature^30,31^. Various level-based indices have been employed from the fields of environmental acoustics and acoustic ecology to explain the frequency content and characterize the temporal changes of the audio signal with the aim of assessing noise pollution levels and detecting presence of species^32,33^. These studies, usually based on long-term measurements by sensor networks deployed in PNAs and noise propagation models, rely on sound pressure level (SPL)-based indices, such as L_Aeq_ and L_den_ for cumulative noise exposure over a whole day. These are often calculated at the sensor node but raw audio can also be collected for subsequent analyses. Despite numerous studies showing evidence that audio signal analysis-only approach cannot explain perceptual and behavioural outcomes of the human experience in sufficient detail^34–36^ the number of studies employing the ISO 12913 Acoustics: Soundscape framework in PNAs or similar mixed methods approaches is extremely limited.

Ferrari et al.^37^ have found that anthropogenic sounds have negative influence on the perceived recreational quality in PNAs. The same holds for a noise level increase beyond 38 dBA^37^ which is a very conservative value compared to urban areas where a typical threshold for acoustic comfort is considered to be around 65 dBA^38^. This implies that the increase in popularity of a site and the number of visits can have an adverse effect, not only on the natural habitats but on the visitors themselves by further contributing to noise pollution. This implies a role of the context as an understanding of what a place people find themselves in is and what it means to them.

### Measuring soundscapes: the ISO 12913 series

The environmental acoustic metrics required by the Parts 2 and 3 of the ISO/TS 12913^8,9^ include the psychoacoustic measurements, or sound quality metrics, developed by Zwicker & Fastl^39^ initially for the purpose of evaluating auditory characteristics of machinery and products, and defined by the respective international standards as shown in Table 1. Regarding the qualitative data, in its Annex C, the ISO/TS 12913-2 features three different tools: questionnaire approach (Method A and Method B questionnaires) or the narrative interview approach (Method C). Method B questionnaire was designed for use in soundwalks, while the Method A can be deployed as either a traditional on-site survey, a soundwalk or in laboratory settings. It has been shown in the past 6 years since the publishing of the ISO/TS, that the Method A has been the most widely accepted approach^40^ and is the one adopted in this study. It features the assessment of the perceived affective quality (PAQ), based on the circumplex model featuring a two-dimensional perceptual space defined by the orthogonal main axes, labelled as Pleasant and Eventful^34^ as shown in this study’s Results (Figs. 2 and 3).

**Table 1:** Environmental acoustic measures required and recommended per ISO/TS 12913-2^8^.

Measurement	Description	Calculation standard
Minimum required per ISO/TS 12913 -2^8^		
L_Aeq, T_	A-weighted equivalent continuous sound pressure level for the period T, where A-weighting stands for filtering high and low frequency ends following the A-weighting curve, providing a representation more similar to human hearing because, unlike measurement microphones that feature “flat frequency response”, humans perceive “middle range frequencies” (around 1 kHz) louder than higher and lower frequencies of the same SPL value.	ISO 1996-1, IEC 61672-1^41,42^
L_Ceq, T_	C-weighted equivalent continuous sound pressure level, where C-weighting stands for filtering high frequency end following the C-weighting curve. Compared to A-weighting, the C-weighting preserves the low frequency information.	ISO 1996-1, IEC 61672-1^41,42^
L_AF5, *T*_	Percentage exceedance level – 5% of the time interval *T*, approximates sound events	ISO 1996-1, IEC 61672-1^41,42^
L_AF95, *T*_	Percentage exceedance level – 95% of the time interval *T*, approximates background noise	ISO 1996-1, IEC 61672-1^41,42^
*N*_5_	Loudness exceeded in 5% of the time interval	ISO 532-1^43^
*N*_95_	Loudness exceeded in 95% of the time interval	ISO 532-1^43^
*N*_*rmc*_	Root mean cubed loudness	ISO 532-1^43^
Recommended per ISO/TS 12913- 2^8^		
*S*	Sharpness, representing the sensation of timbre with emphasis on high frequencies	DIN 45,692^44^
*T*	Tonality, representing the sensation of timbre and whether a sound consists of tonal components or broadband sound	ECMA-74^45^
*R*	Roughness, representing sounds modulated at higher modulation frequencies	
*F*	Fluctuation strength, representing sounds modulated at low modulation frequencies	
Additional measurements considered^46^		
L_Ceq, *T*_ - L_Aeq, *T*_	Difference between the L_Ceq, *T*_ and L_Aeq, *T*_, revealing the equivalent continuous sound pressure level for the low frequency part of the spectrum	ISO 1996-1, IEC 61672-1^41,42^
L_AF5, *T*_ – L_AF95, *T*_	Difference between the L_AF5, *T*_ and L_AF95, *T*_, revealing the relation between single sound events and the background	ISO 1996-1, IEC 61672-1^41,42^

Soundwalk is the recommended method for obtaining human responses based on a participatory listening walk along a (predetermined) route, featuring a number of listening stops – measurement points and a number of participants gathered at the location for the specific purpose of the soundwalk^8^. However, most of the research that fed into the ISO 12913 Acoustics – Soundscape series was conducted on urban environments, with urban setting in mind where a tolerance to certain noise sources is perhaps an integral part of the urban soundscape aesthetics. Mlynarczyk & Wiciak^47^ have compared urban soundscape data^48^ with the perceptual data from a national park in laboratory conditions using the “virtual soundwalk approach”^49^showing the majority of recordings from the national park being mapped in the pleasant and uneventful space. Conversely, while there is a growing number of studies exploring soundscape pleasantness and eventfulness in various urban settings and laboratory conditions^40^to the best of authors’ knowledge, there are no available studies conducting soundscape investigations in PNAs in a way compliant with the ISO recommendations for assessments in situ.

### Study objectives

This study, based on the five expeditions conducted by the *Silenzi in Quota* initiative^50,51^ aims to address the research gap identified by providing evidence about the application of the ISO 12913 framework in PNAs and deepening the understanding of the effect of environmental sounds on human perception in PNAs. This is achieved by gathering perceptual in situ data at locations hard-to-reach and investigating the associations between the key (psycho)acoustic metrics and perceptual measurements. The manuscript has been structured in a way to provide answers to the following Research Questions:

1. How are the perceptual, context-related measurements (perceived sound sources dominance and overall perceived visual quality of the environment) influencing the perceived soundscape quality (pleasantness and eventfulness) in PNAs? (RQ1)
2. What are the (psycho)acoustic features influencing perceived soundscape quality (pleasantness and eventfulness) in PNAs? (RQ2)

## Results

### Acoustic measurements

The range of acoustic conditions observed across all the measurement points are described in Table 2 in terms of both acoustic and psychoacoustic variables. The investigated sites ranged from very quiet to rather loud environments, with an overall range of nearly 45 dB. The full details on all the acoustic measurements taken, per site, are available at the online repository^52^.

**Table 2:** The range of acoustic conditions across all the measurement points, based on five expeditions in four PNAs (five in Italy, one in the United Kingdom (UK)) and 23 audio recordings.

Psycoacoustic measure	Min.	Max.	Mean	Median	St. dev.
L _Aeq, T_	31.2	76.1	48.4	47.8	11.9
L_Ceq, T_–L_Aeq, T_	0.4	14.6	3.8	2.7	3.4
L_AF5,T_– L_AF95,T_	1.0	23.1	8.2	7.0	5.5
N_5_/N_95_	1.09	3.85	1.89	1.78	0.69
N _rmc_	1.71	37.30	7.51	5.36	8.63
S	1.01	3.30	1.91	1.84	0.46
R	0.013	0.061	0.025	0.023	0.010
F	0.002	0.066	0.019	0.010	0.018
T	0.015	0.392	0.113	0.067	0.107

### Perceptual measurements

**Fig. 1:**
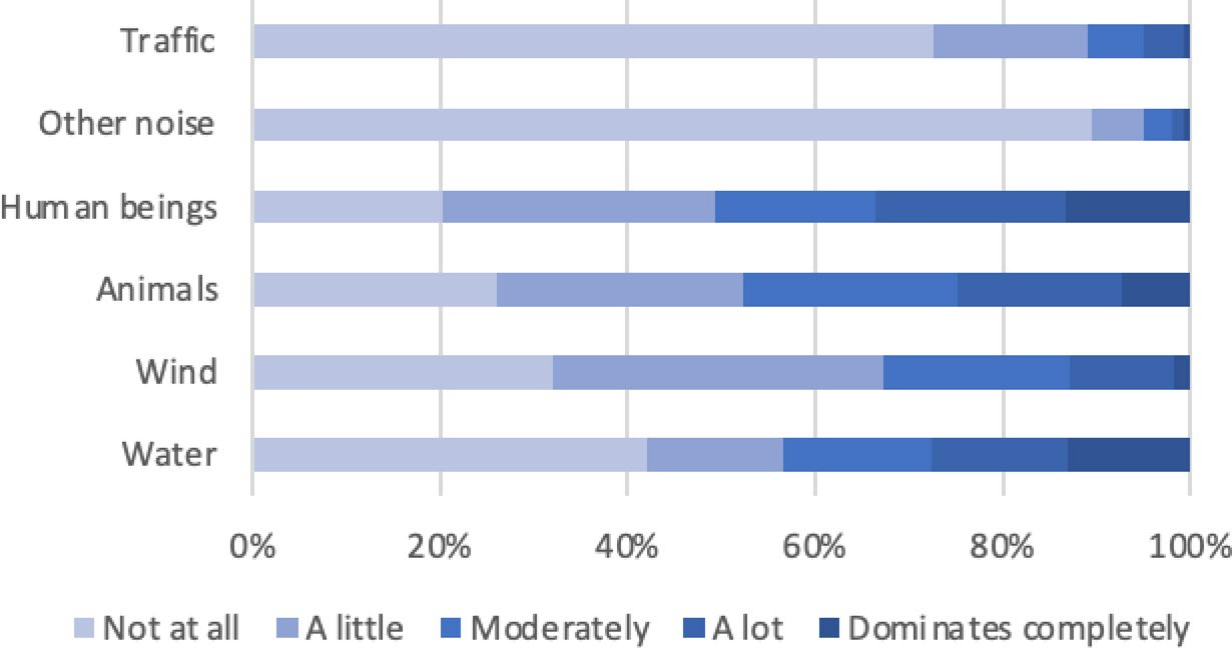
Perceived dominance of different sound types, based on a varying number of observations (*N* = 435–439) across 28 listening stops.

The perceived dominance of sound sources is illustrated in Fig. 1, highlighting the character of the study locations covered by the soundwalks. These areas are characterized by the dominance of human sounds (e.g., voices, moderately, a lot, or completely dominating in 51% of cases, overall N: 435) and natural sounds, such as those produced by animals (dominating in 48% of cases, N: 438), water (44%, N: 439), and wind (33%, N: 435). Traffic noise and other noises (e.g., sirens or industrial sounds) are generally not heard (traffic: moderately, a lot, or completely dominating in 11% of cases, N: 439; other noise: 5%, N: 436).

Regarding the visual landscape, the evaluations are, as expected, very positive. In 94% of the evaluations visual landscape is rated as good or very good (N: 439).

### Relationship between sound sources dominance, overall visual quality, soundscape pleasantness and eventfulness (RQ#1)

The results of LMM1_P for ISO Pleasantness show a significant effect of the dominance of traffic noise (χ^2^ 166 (4) = 15.105, *p* = 0.005, η^2^ = 0.14 the degrees of freedom are reported in brackets), other sounds (e.g., sirens, construction, industry, loading of goods) (χ^2^ (1) = 4.036, *p* = 0.045, η^2^ = 0.04), sounds generated by other human beings (χ^2^ (1) = 53.663, *p* < 0.001, η^2^ = 0.49), water sound (χ^2^ (1) = 4.327, *p* = 0.037, η^2^ = 0.04), and the quality of the visual landscape (χ^2^ (1) = 21.693, *p* < 0.001, η^2^ = 0.20). Specifically, greater ISO pleasantness is associated with less traffic noise, construction noise and human voices, more dominant sound produced by water features, and better landscape quality (see Table 3). Gender, age, mountain sports habits, dominance of animals, and wind are not found to be significantly associated with the ISO Pleasantness of the sound environment.

**Table 3:** Results of LMM1_P and LMM1_E models reporting estimates, *p*-values and VIF/GVIF values for each fixed effect within the computed models for ISO Pleasantness and ISO Eventfulness.

Response variable	Fixed effect	Estimate	Standard error	*p*-value	VIF/GVIF
ISO Pleasantness	Q1	0.045	0.03229	0.159	1.020
	Q2	0.002	0.0015	0.182	1.013
	Q3	− 0.051	0.04318	0.241	1.025
	**Q4.1**	**− 0.512**	**0.143845**	< 0.001***	**1.014**
	**Q4.2**	**− 0.053**	**0.026516**	0.045*	**1.022**
	**Q4.3**	**− 0.122**	**0.016779**	< 0.001***	**1.033**
	Q4.4	0.030	0.015372	0.051	1.039
	Q4.5	0.019	0.014416	0.176	1.031
	**Q4.6**	**0.029**	**0.014108**	0.038*	**1.027**
	**Q8**	**0.101**	**0.021785**	< 0.001***	**1.014**
ISO Eventfulness	Q1	5.494e−05	0.00134	0.967	1.021
	Q2	0.03555	0.0272	0.201	1.017
	Q3	− 3.227e−03	0.032	0.930	1.027
	**Q4.1**	**3.337e−01**	**0.01167** **0.0155**	0.031*	**1.014**
	Q4.2	1.861e−02	0.0282	0.510	1.019
	**Q4.3**	**1.521e−01**	**0.0177**	< 0.001***	**1.038**
	Q4.4	2.252e−02	0.0163	0.167	1.042
	Q4.5	− 1.239e−02	0.0151	0.410	1.034
	**Q4.6**	**3.150e−02**	**0.0150**	0.037*	**1.029**
	Q8	2.179e−02	0.0226	0.335	1.016

Fixed effects codes represent questions form the survey and are described in Table 8. Significance codes for the *p*-values: ***< 0.001, **< 0.01, *< 0.05. Number of observations = 411.

Significant values are shown in bold.

As regards ISO Eventfulness, LMM1_E indicates a significant main effect of the dominance of traffic noise (χ^2^ (4) = 7.203, *p* = 0.045, η^2^ = 0.03), and human voices (χ^2^ (1) = 74.099, *p* < 0.001, η^2^ = 0.91), and water sounds (χ^2^ (1) = 4.390, *p* = 0.036, η^2^ = 0.05). Higher eventfulness is associated with more dominant traffic noise, human sounds and water sounds (see Table 3).

**Fig. 2:**
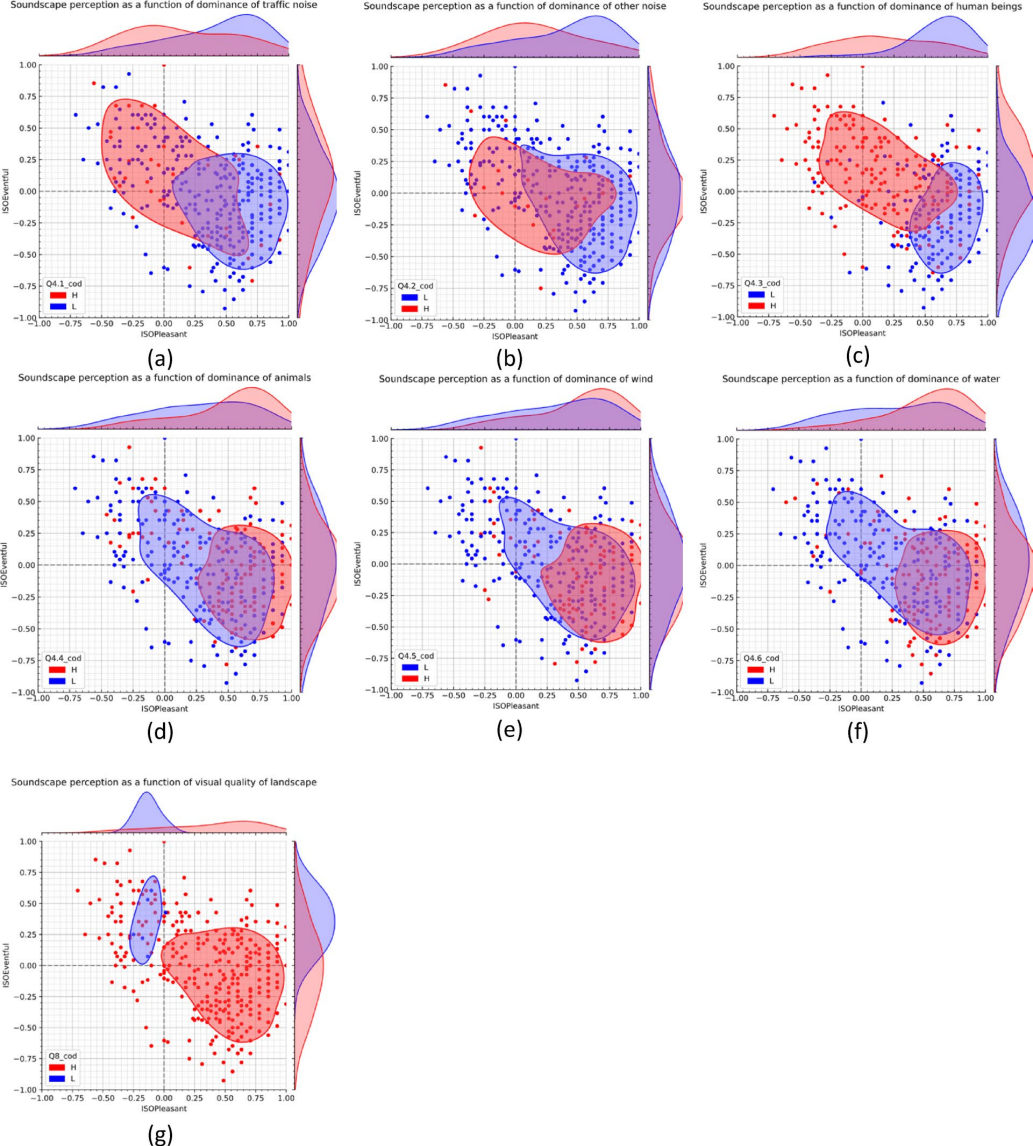
Comparison of soundscapes based on the dominance of (**a**) traffic noise, (**b**) other noise, (**c**) human beings, (**d**) animals, (**e**) wind, (**f**) water sounds, and (**g**) quality of landscape. The curves represent the 50th percentile contour, and the bivariate distributions of ISO Pleasantness and ISO Eventfulness are plotted on the two axes. "L" represents low dominance (not at all, a little) or poor quality (very bad; bad) group, while "H" represents the high dominance (moderately, a lot, dominates completely) or high quality (neither good nor bad, good; very good) subsample.

The soundscape assessments are represented in Fig. 2 with evaluations divided into two groups based on the perceived dominance of sounds (low or high, for traffic noise in Fig. 2a, other noise in Fig. 2b, human sounds in Fig. 2c, animal sounds in Fig. 2d, wind sounds in Fig. 2e, and water sounds in Fig. 2f) or the perceived quality of the landscape (low or high, as in Fig. 2g).

### Relationship between the (psycho)acoustic features and soundscape pleasantness and eventfulness (RQ#2)

The single-parameter models (LMM2_P to LMM10_P) for ISO Pleasantness show a significant association with the A-weighted continuous equivalent sound pressure level L_Aeq, T_ (χ^2^ (1) = 6.789, *p* = 0.009), L_AF,5_ - L_AF,95_ (χ^2^ (1) = 8.765, *p* = 0.003), tonality (χ^2^ (1) = 27.332, *p* < 0.001), and fluctuation strength (χ^2^ (1) = 27.230, *p* < 0.001). Higher sound levels, sound level variation over time, tonality, and fluctuation strength values correspond to less pleasant and more annoying soundscapes (see Table 4).

**Table 4:** Results of LMM models reporting estimates, and *p*-values for each fixed effect within the computed models for ISO Pleasantness and ISO Eventfulness.

Response variable	Model number (*n*.)	Fixed effect	Estimate	Standard error	*p*-value
ISO Pleasantness	**LMM2_P**	**L**_**Aeq, T**_	**− 0.012**	**0.0047**	0.017*
	LMM3_P	L_Ceq, T_–L_Aeq, T_	− 0.003	0.018	0.845
	**LMM4_P**	**L**_**AF5**,***T***_**-L**_**AF95**,***T***_	**− 0.029**	**0.0098**	0.007**
	LMM5_P	N _rmc_	− 0.008	0.0072	0.273
	LMM6_P	N_5_/N_95_	− 0.154	0.0088	0.096
	**LMM7_P**	**T**	**− 2.241**	**0.42872**	< 0.001***
	LMM8_P	S	− 0.004	0.1486	0.980
	LMM9_P	R	− 4.303	5.2021	0.418
	**LMM10_P**	**F**	**− 14.009**	**2.6846**	< 0.001***
ISO Eventfulness	**LMM2_E**	**L**_**Aeq, T**_	**0.015**	**0.0033**	< 0.001***
	LMM3_E	L_Ceq, T_–L_Aeq, T_	− 0.005	0.00157	0.763
	**LMM4_E**	**L**_**AF5**,***T***_**-L**_**AF95**,***T***_	**0.020**	**0.0088**	0.036*
	**LMM5_E**	**N**_**rmc**_	**0.014**	**0.0060**	0.030*
	LMM6_E	N_5_/N_95_	0.124	0.07915	0.132
	**LMM7_E**	**T**	**1.943**	**0.3666**	< 0.001***
	LMM8_E	S	0.189	0.1228	0.141
	**LMM9_E**	**R**	**9.400**	**4.2124**	0.036*
	**LMM10_E**	**F**	**11.108**	**2.51845**	0.001**

Significance codes for the *p*-values: ***< 0.001, **< 0.01, *< 0.05. Number of observations = 389 (some missing values were detected for Baita Segantini and Rifugio Capanna Cervino).

Significant values are shown in bold.

Regarding the modelling of ISO Eventfulness, the single-parameter models (2 to 10) exhibit a significant correlation with the A-weighted continuous equivalent sound pressure level L_Aeq, T_ (χ^2^ (1) = 20.328, *p* < 0.001), L_AF,5_ - L_AF,95_ (χ^2^ (1) = 8.7652, *p* = 0.003), loudness (χ^2^ (1) = 5.6013, *p* = 0.018), tonality (χ^2^ (1) = 28.068, *p* < 0.001), roughness (χ^2^ (1) = 4.979, *p* = 0.026), and fluctuation strength (χ^2^ (1) = 19.454, *p* < 0.001). Specifically, more eventful soundscapes are associated with higher sound levels, level variation over time, loudness values, tonality, roughness, and fluctuation strength values.

**Fig. 3:**
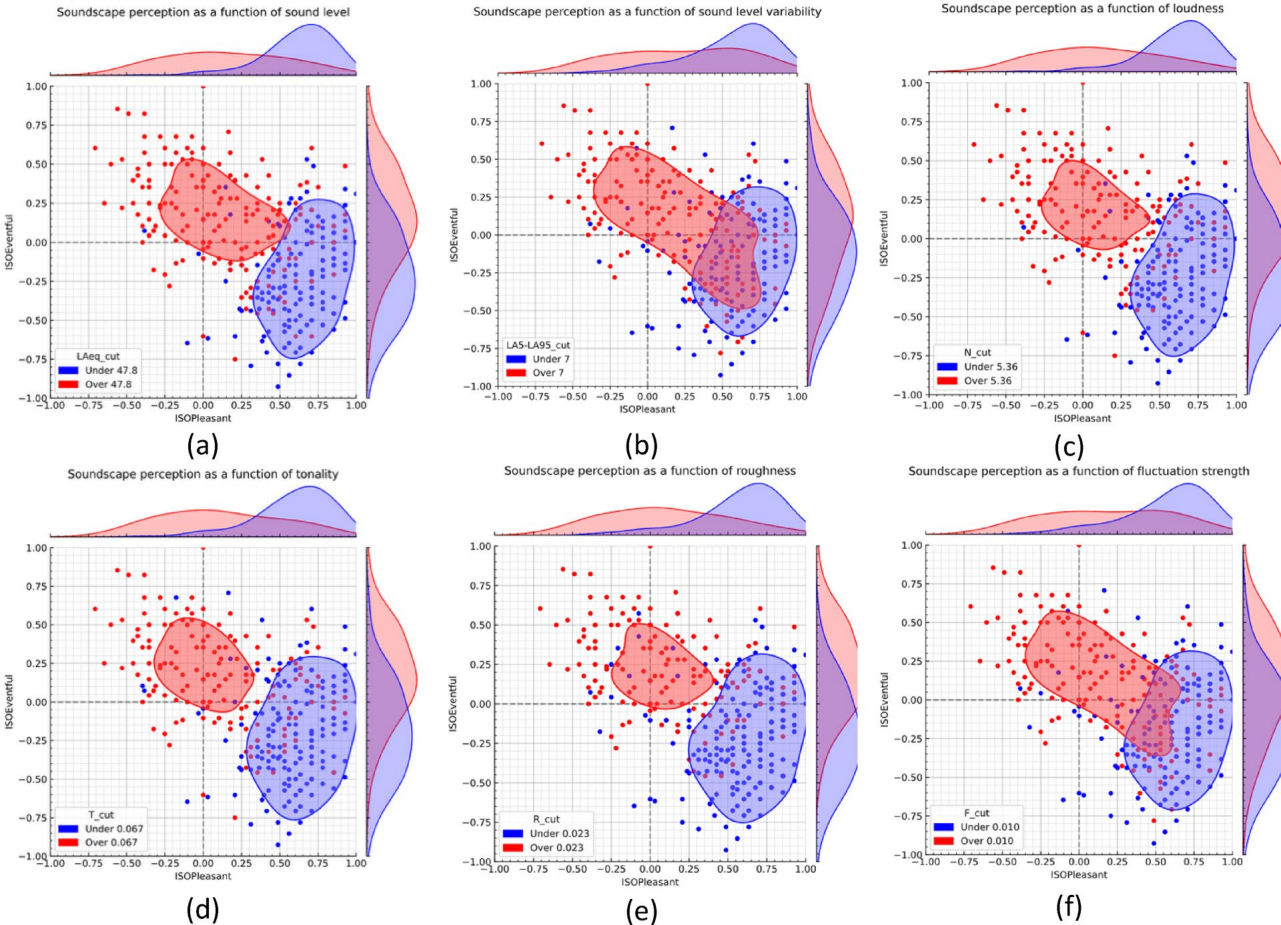
Comparisons of soundscapes based on the values of (**a**) L_Aeq_, (**b**) L _AF5,T_-L _AF95,T_, (**c**) N_rmc,_ (**d**) *T*, (**e**) *R* and (**f**) *F*. The dataset was divided into two subsamples based on the median value of the three parameters. The curves represent the 50th percentile contour, and the bivariate distributions of pleasantness and eventfulness are plotted on the two axes.

The effects of sound pressure level (Fig. 3a), sound level variability (Fig. 3b), loudness (Fig. 3c), tonality (Fig. 3d), roughness (Fig. 3e) and fluctuation strength (Fig. 3f) on soundscape are illustrated in Fig. 3, where the dataset is divided into two sub-samples based on the median value of each (psycho)acoustic variable (see Table 2). This allows for a comparison of soundscape contours (i.e., the curves representing the 50th percentiles) according to high vs. low levels of sound, loudness, and tonality. We can notice that responses scoring high in these psychoacoustic values are generally neutral in terms of pleasantness and more eventful. In quieter locations, with less sound level variation, lower roughness, tonality and fluctuation strength the soundscape contours are generally positioned in an area of greater pleasantness and lower eventfulness, thus resulting in a calmer soundscape. Moreover, it can be noticed that the two soundscape contours based on the median value of tonality are particularly distinct and separate, clearly defining an eventful zone with high tonality values and a calm zone with low tonality.

The AIC, the R_m_^2^ and R_c_^2^ coefficients are reported in Table 5, with lower AIC values corresponding to higher predictive power of the model, and higher R^2^ associated to higher proportion of variance in the dependent variable explained by the independent variables.

**Table 5:** AIC, marginal and conditional R^2^ of the LMM for each dependent variable.

Response variable	Model number (*n*.)	AIC	*R* ^2^_marginal_	*R* ^2^_conditional_
ISOPpleasantness	LMM1_P	50.241	0.387	0.711
	LMM2_P	112.25	0.13	0.71
	LMM3_P	118.7	0.00	0.73
	LMM4_P	111.21	0.16	0.71
	LMM5_P	117.41	0.03	0.73
	LMM6_P	115.57	0.07	0.71
	LMM7_P	100.06	0.31	0.61
	LMM8_P	118.74	0.05	0.61
	LMM9_P	117.93	0.08	0.57
	LMM10_P	101.43	0.28	0.61
ISO Eventfulness	LMM1_E	92.043	0.269	0.577
	LMM2_E	98.436	0.25	0.59
	LMM3_E	113.783	0.07	0.71
	LMM4_E	108.952	0.09	0.58
	LMM5_E	108.399	0.11	0.59
	LMM6_E	111.317	0.06	0.58
	LMM7_E	94.387	0.34	0.72
	LMM8_E	112.061	0.00	0.73
	LMM9_E	109.011	0.01	0.72
	LMM10_E	98.839	0.35	0.73

For both ISO Pleasantness and ISO Eventfulness, perceptual models (LMM1_P and LMM1_E) outperform psychoacoustic models, resulting in considerably lower AIC values, especially for pleasantness. Among psychoacoustic ones, single-parameter models based on tonality (LMM7_P) and fluctuation strength (LMM10_P) are the most effective for predicting pleasantness, corresponding to lower AIC values. Regarding eventfulness, the tonality parameter (LMM7_E) has a similar performance in predicting eventfulness compared to perceptual models (i.e., within 2 AIC units).

Interestingly, the marginal (R^2^_m_) coefficients of determination are significantly lower than the conditional (R^2^_c_) ones for each model. This outcome suggests that a greater proportion of the variance was accounted by random effects related to the experimental design (i.e., participants, locations nested in sites) rather than by fixed effects (i.e., perceptions and measurements).

## Discussion

### Interpretation

#### RQ1—How are perceived sound source dominance and overall perceived visual quality of the environment influencing the perceived soundscape pleasantness and eventfulness in PNAs?

The effects of the perceived sound source dominance and the overall perceived visual quality of the environment on the ISO Pleasantness and ISO Eventfulness were explored using the questionnaire results only. The questionnaire item investigating the composition of natural sound source type, from the ISO/TS 12913-2^8^, was expanded into additional three questions to capture animal, wind-driven and water sounds. This, more detailed sound source dominance questionnaire has revealed that different types of natural sounds contribute to ISO Eventfulness in different ways. Namely, the animal (Q4.4) and wind (Q.4.5) sounds showed no significant effect, but dominance of water sounds (Q4.5) exhibited a positive correlation with ISO Eventfulness. This implies that in natural areas, a more detailed sound source type appraisal is useful.

Watts et al.^53^ explored the combined effect of the acoustic environment, as captured by microphone-based sensors, together with the content of environmental sounds and the context they are experienced in, and demonstrated that in urban environments the presence of visible vegetation can increase human tolerance to noise^53^. In urban parks, it was found that higher human presence under a certain threshold would increase both auditory and visual satisfaction with an environment^54^. In “more extreme” urban environments, such as central business districts, the dominance of human sounds has also been found to be associated with higher ISO Pleasantness^55^. However, this study indicated that an increase in dominance of human sounds leads to a decrease in ISO Pleasantness. This difference is most likely driven by the expectations people have when visiting PNAs, which are different than in cities, both in urban parks and central business areas. Visiting a natural site is an effort implying both planning and financial cost, aimed at escaping everyday urban environments and achieving a connection with nature. Not meeting such expectations likely results in a feeling of disappointment. Indeed, this is similar to the findings by Pérez-Martínez et al.^56^ who reported a decrease in pleasantness associated with human sounds at a cultural heritage site with a strong (over)tourism component in Granada, Spain.

Papadakis et al.^57^ have looked into the influence of different expectations driving ISO Pleasantness and ISO eventfulness, namely the residence and participants’ background as a proxy for familiarity with certain urban acoustic environments. Indeed, familiarity was the third dimension, following valence and arousal, recognized by Axelsson et al.^34^. In this study, Q3 (Do you often (at least once a month) practice mountain sports? ) was used as the proxy for familiarity with natural areas similar to the ones investigated but no effect was found through the analysis. This is in line with Yang et al.^58^ who looked at the effect of tourism and showed that both residents and visitors display equal appreciation of natural sounds.

The questionnaire-based models LMM1_P and LMM1_E demonstrated the highest predictive power, as assessed by observing the AIC, the R_m_^2^ and R_c_^2^ coefficients (see Table 5). This speaks for the potential of using crowd-sourced questionnaire data from soundwalks or equivalent smartphone-based applications, such as^59,60^ over traditional sound level monitoring stations for predicting soundscape quality. This is in line with other similar studies comparing the physiological and psychophysical models^61^. Additionally, the higher LMM1_P and LMM1_E performance implies the benefit of accounting for the types of sources which are audible, highlighting the potential application of machine learning-based automatic source recognition methodologies^62^ to characterize soundscapes in natural areas. While the focus of this study was to observe the effect of human activity on soundscape of PNAs, this finding is in line with other studies investigating the effect of traffic noise on annoyance where perceptual models tend to outperform the ones based on psychoacoustic features only^63^. The LMM1_P performed significantly better than the LMM1_E, confirming the higher difficulty in predicting eventfulness/content compared to pleasantness/comfort already found for urban^34^ and indoor soundscapes^64^.

Regarding the effect of the visual context, it is important to note that the distribution of Q8 (Overall, how would you describe the present surrounding visual environment?) responses is skewed towards very positive. This was expected, given that all the soundwalks took place in areas that are tourist attractions. A positive correlation was found between the overall visual quality and ISO Pleasantness, in line with the findings from other studies in urban parks where it was found that a more attractive natural scene can improve soundscape^65^. However, the number of negative soundscape quality assessments in this study still proves that not even the very high visual attractiveness of a site is sufficient to ensure a high-quality natural environment and its soundscape.

#### RQ2—What are the (psycho)acoustic features influencing perceived soundscape pleasantness and eventfulness in PNAs?

The (psycho)acoustic measurements that displayed the strongest effect on the ISO Pleasantness were *T*, *F*, L _AF5,*T*_-L _AF95,*T*_ and L_Aeq, *T*_. The strongest effect on ISO Eventfulness were *T*, *F*, L _Aeq, T_, *R* and N _rmc_. Tonality emerged as the main psychoacoustic feature affecting both perceived soundscape pleasantness and eventfulness. The model reveals negative coefficients for ISO Pleasantness (i.e., higher tonality leads to higher annoyance) and positive coefficients for ISO Eventfulness; hence, following the structure of the soundscape circumplex model, one could infer that higher tonality in the acoustic environment of PNAs included in this study is related to higher perceived sense of chaos (i.e., a soundscape that features negative ISO Pleasantness and positive ISO Eventfulness can be defined as chaotic).

At the sites investigated in this study, higher tonality (between 0.1 and 0.4 tu) seems to be associated with higher perceived dominance of human sounds (voices from people in this case), as shown in Table 6. This is in line with findings by Yang & Kang^66^ where it was observed that high presence of human speech can result in tonality around 0.1 tu. While they^66^ observed birdsong to be usually more tonal (between 0.5 and 0.8 tu), in this study, it (Q4.4) didn’t result in tonality higher than 0.4 tu. It is important to note that such psychoacoustic measures are highly dependent on the overall acoustic context and all the measurements made are performed on the samples of complex environments containing a multitude of sound sources in random relationship, including their random relative distances. In urban context due to the presence of more dominant anthropic sound sources (e.g., traffic noise, mechanical sounds), not present in PNAs, human voices do not stand out as particularly tonal sound sources as they are “masked” by the urban noise background. In such context tonality often reaches higher values, above 0.4 tu in cases of acoustic environments containing anthropogenic sounds such as church bells or music^66,67^. Therefore, the range of tonality values observed in this study still falls in the ‘low tonality range’, demonstrating the importance of considering context when assessing complex auditory environments.

**Table 6:** Spearman correlation coefficients and p-values between the psychophysical measures and perceived sound source type dominance.

Questionnaire item	L _Aeq, T_	L_Ceq, T_–L_Aeq, T_	L _AF5,T_–L _AF95,T_	*N*_rmc_	*N*_5_/*N*_95_	*T*	*S*	R	*F*
Q4.1 (traffic noise)	0.27**	0.25**	0.33**	0.27**	0.33**	0.24**	− 0.34**	0.33**	0.23**
Q4.2 (other noise)	0.11*	0.19**	− 0.03	0.11*	− 0.02	0.16**	− 0.12*	0.06	0.11*
Q4.3 (human sounds)	0.45**	0.28**	0.56**	0.45**	0.44**	0.75**	− 0.25**	0.37**	0.75**
Q4.4 (animal sounds)	− 0.39**	− 0.05	0.05	− 0.37**	0.06	− 0.07	0.14**	− 0.43**	− 0.15**
Q4.5 (wind sounds)	− 0.24**	0.13*	− 0.02	− 0.21**	0.03	0.02	− 0.21**	− 0.18**	− 0.12*
Q4.6 (water sounds)	− 0.05	− 0.53**	− 0.42**	− 0.05	− 0.44**	− 0.54**	0.47**	− 0.05	− 0.47**

Significance codes for the* p*-values: ***< 0.001, **< 0.01, *< 0.05.

Significant values are shown in bold.

Other studies looking at the effects of psychoacoustic measures on ISO Pleasantness and ISO Eventfulness performed in urban context, including large urban parks, have found a strong effect of loudness, sharpness and L_Aeq_, while the effect of tonality was noted but was found to be less important than in this study^46^.

While the association between the dominance of human sounds and annoyance is clear, it is important to note that the human sounds are in fact the most frequent sound source type observed across the sample (Fig. 1). Indeed, up to a certain threshold, Ednie et al.^68^ have found that urban visitors still prefer to experience urban noises in protected areas. Taking tonality as a proxy for human sound presence (see Table 6), we can derive threshold values for ISO Pleasantness and ISO Eventfulness based on linear regression models. These are *T* = 1.248 tu for ISO Pleasant (ISO Pleasantness = 42.653 + 34.17 *T*, *p* < 0.001, R^2^_adj_ = 0.53) and *T* > 0.021 tu for ISO Eventful (ISO E = 0.503 + 23.777 T, *p* < 0.001, R^2^_adj_ = 0.45). Therefore, a tonality threshold indicating chaotic soundscapes (i.e., both unpleasant and eventful) in PNAs could be as low as 0.021 tu.

Fluctuation Strength (*F*) is a psychoacoustic measure indicating the presence of low modulation frequencies in audio signal (around 4 Hz). Typically, *F* is associated with the presence of sounds sources such as the wind farm noise, but also human speech^69^. In this study it was tied to human sounds, similarly as the tonality. This is not uncommon^66^and it is a feature that was found to be positively associated with ISO Eventfulness and negatively associated with ISO Pleasantness in urban context as well. Based on linear regressions on collected data, a fluctuation strength higher than *F* > 1.78 vacil is likely to be causing negative ISO Pleasantness (ISO Pleasantness = 40.283 + 34.183 F, *p* < 0.001, R^2^_adj_ = 0.53), while an indicative threshold for ISO Eventful is 0.011 vacil (ISO Eventfulness = 0.269 + 23.796 F, *p* < 0.001, R^2^adj = 0.45). Therefore, a fluctuation strength indicating chaotic soundscapes in PNAs would be* F* > 0.011 vacil. On the other hand, Pheasant et al.^70^ have reported thresholds of L_Amax_ < 55 dB and L_Aeq_ < 42 dB to achieve a high tranquillity score. These findings were achieved in laboratory settings, based on 32s-long audio samples. While this study is not attempting on making a direct connection between the dimensions present in the soundscape circumplex model and the tranquillity construct, in the Fig. 3a) it can be observed that a threshold for a calm and pleasant soundscape lies somewhere above L_Aeq_ < 48 dB. This can probably be explained due to the following facts: (1) this study was conducted on-site, where a wider range of sound sources is present in their ecologically true setting, (2) dominance of water sounds proved to be associated with pleasant soundscape, yet a number of sites close to waterfalls that were captured in this study feature L_Aeq_ values > 42 dB. Most importantly, our study revealed tonality to be a better perceptual predictor than L_Aeq_.

A practical implication for monitoring and assessment of soundscape in PNAs is that both subjective and objective measurements are necessary for accurate characterisation following the ISO 12913 framework^7–9^, while the ability to accurately monitor tonality and fluctuation strength on-site is more important than controlling sound pressure levels only. Moreover, applying management policies to improve sound-related behaviour of the visitors, .i.e. lowering their “noise footprint”^71^is crucial for ensuring positive experience of natural areas for the visitors, such as the one demonstrated by Stack et al.^72^.

### Limitations and future pathways

PNAs are expected to feature a very high variability in human presence from overcrowded beauty spots and the associated walking paths and roads, to the parts that almost never get visited. Both types of sites can suffer from anthropogenic noise. This study is biased towards capturing the effect of overcrowding. However, even in such conditions, recruitment and obtaining consistent data can pose a challenge when compared to urban conditions. Method A presented in the Annex C of the ISO/TS 12913-2^8^ was considered to provide a solid solution to characterize soundscape in PNAs using subjective questionnaire data and objective acoustic measurements. The large spread of responses within the two-dimensional circumplex space, and the large spread of measured (psycho)acoustic indices confirm that such conditions can be captured via this type of soundwalks.

However, it must be noted that conducting a soundwalk in a remote area brings up challenges related to the size of the area that can be covered, duration of the walk that is manageable to most participants, number of participants that cannot be too large before starting to bias the results and that the data are limited to the accessible hiking paths.

While it can be argued that leading a soundwalk with a group of participants represents a less ecologically valid approach to characterizing soundscapes due to the bias of ‘participants’ presence’ and the fact that participants at the last stop are likely more attentive to the whole procedure than at the first stop, the authors argue that this approach still ensures the following key advantages compared to different sampling strategies, such as the one employed by Ferrari et al.^37^: (1) all the ratings from each listening stop relate to same environmental conditions, (2) a number of questionnaire responses can be collected in one day characterising a hiking path of up to 12 km length. Also, we believe our characterization is relevant for the typical hiking experience (in a group), as lone hiking is nor typical, not recommended for safety purposes. The bias of experiencing a site within a group of people, compared to an experience of a lone visitor, was also not considered significant as it’s not uncommon to encounter other visitors in these popular mountain environments.

The questionnaire tool chosen for this study based on its popularity for soundscape research^40^ was developed by using sample locations characteristic for urban environments. Studies exploring the applicability of that tool for use in different context, such as indoor residential environment, have suggested some modifications to the attributes used but have confirmed the underlying structure of a valence-arousal circumplex model. Therefore, it was considered adequate for this study and has provided meaningful results that can be interpreted in a logical way. However, as most of the responses are gathered along the diagonal between chaotic and calm soundscapes, future research might be needed to properly address the state of excitement while exploring wilderness, which might be different from calm, pleasant or vibrant dimensions. Moreover, before the establishment of the ISO 12913 series, tranquillity was one of the perceptual constructs that has received more consistent attention by the research community when it comes to using mixed methods approaches to explain perceptual outcomes of exposure to an environment. Herzog and Barnes used it to characterize quietness and quite places^73^. It was redefined and extensively studied in both urban and natural areas by Watts and Pheasant^24,70,74,75^. Contextual features, such as the presence of visual natural features in a scene, were established as key factors contributing to the construct^70^ but the association with quietness and calmness was kept. So another, complementary construct, aimed at providing a more detailed characterization of natural settings introduced by Pheasant and Watts was wildness^23 ^and it included considerations of felt remoteness and naturalness^23,76^ which provides a possible direction towards further explorations of the optimal attributes for assessing soundscape in PNAs.

Negligible number of participants used the opportunity to provide more information in the open-ended Q9 (Do you have any comment on this listening point? ). This is most likely because writing during a soundwalk in such locations could be considered impractical, so it speaks for the use of box-ticking questionnaires. For that reason, the use of short, structured interviews after the soundwalk sessions should be considered in future work to provide richer data sets and more opportunities to interpret the questionnaire data accurately.

Regarding the statistical analyses strategy, the two multivariable models (LMM1_P and LMM1_E) were built to evaluate the effects of the perceptual, questionnaire-based variables (RQ1). However, multiple models were built for different (psycho)acoustic sensor-based variables (RQ2). We observed a certain degree of collinearity, contributing to the decision not to build a single model featuring all the variables, nor a model with a subset of them, but rather to explore the effect of the various (psycho)acoustic variables independently, in an exploratory manner. This was done for the sake of interpretability and to avoid standard error inflation, despite not accounting for the shared variance between the predictors. Moreover, such a choice was considered suitable for the exploratory nature of this study and the aim to evaluate impact of specific variables suggested by the ISO 12913 series^7–9^, taking into account the multidimensional nature of soundscape-driven problems. Indeed, future research, based on a larger data set and featuring a greater variety of environments will enable the development of soundscape predictive models for PNAs, such as the those demonstrated by Mitchell et al. or Ooi et al.^46,77^.

The first soundwalks organised within the Silenzi in Quota initiative took place in 2022. This study reports on the implementation of the ISO 12913 framework not previously tested in mountainous and natural exurban areas to this extent. This work paved the way for future standardisation of soundscape investigations in PNAs and provided evidence for a sustainable approach to visitors’ numbers and behaviour. The importance of investigating influence of exurban context on soundscape has been highlighted together with some limitations of the current ISO 12913 framework when applied in large PNAs. Sound type categories and psychoacoustic features displayed a clearly different pattern than those found in urban context as visitors can easily become the most critical noise source themselves.

## Methods

This study is based on a mixed methods approach featuring the five participatory walks conducted on-site where the subjective data was collected from the participants via a questionnaire tool simultaneously with the short-term environmental acoustic measurements.

### Sites

Five walking routes located within PNAs in the north of Italy (*N* = 4) and Scotland, United Kingdom (*N* = 1) were investigated on a one-session-per-route basis, taking place over a period of 14 months between April 2022 and June 2023. The protection status of the natural areas investigated includes inscription at the UNESCO World Heritage list^78^ and National Park status^79^. The four walking routes in Italy are located within the following three natural areas, all within the zones inscribed to The Dolomites UNESCO World Heritage property: Parco naturale Fanes-Sennes Braies (session Lago di Braies), Parco naturale Panaveggio – Pale di San Martino (sessions Val Venegia and Passo Rolle) and Parco naturale Tre Cime (session Tre Cime di Lavaredo). The walking route in the United Kingdom is within the Cairngorms National Park (session Glen Lui). Throughout the text the five routes will be referred to as per their respective session names in the Table 7, similar to the names chosen in calls for participation via the webpage^50^.

**Table 7:** List of the five soundwalk sessions with route characteristics.

Session	Date	PNA	Level of protection	Length of the walk	Duration of the walk (first to last listening point)	Elevation gain	Lowest and highest point	Number of participants	Number of listening stops	Number of questionnaires collected
Lago di Braies	24th of April 2022	Parco naturale Fanes-Sennes Braies	UNESCO World Heritage	6.1 km	3:45 h	136 m ↑ 136 m ↓	1492 m 1590 m	14	7	98
Val Venegia	19th June 2022	Parco naturale Panaveggio – Pale di San Martino	UNESCO World Heritage	12 km	6:02 h	510 m ↑ 510 m ↓	1676 m 2181 m	6	8	48
Passo Rolle	12th February 2022	Parco naturale Panaveggio – Pale di San Martino	UNESCO World Heritage	3.9 km	2:25 h	226 m ↑ 226 m ↓	1956 m 2182 m	18	4	72
Glen Lui	28th May 2023	Cairngorms National Park	National Parks Authority United Kingdom	12 km	4:50 h	92 m ↑ 92 m ↓	377 m 433 m	25	6	150
Tre Cime di Lavaredo	25th June 2023	Parco naturale Tre Cime	UNESCO World Heritage	9.2 km	2:42 h	303 m ↑ 303 m ↓	2306 m 2451 m	25	3	75

None of the UNESCO documents related to the Dolomites World Heritage Property, available online at the corresponding UNESCO-managed webpage^78^ mention any the following keywords: sound, noise and/or acoustic. The Cairngorms National Park Authority documentation mentions the dominance of natural sounds within the section on Special Landscape Qualities – Visual and Sensory Qualities and provides brief descriptions of the auditory experiences specific to specific types of landscapes within the Park^80^. The section Good Design in National Park^81^ mentions the potential of a well-designed development to reduce overall emissions, including noise, but the good design case studies provide no further details, according to the brief review by the authors.

### Questionnaire

The questionnaire was structured as per the Method A of the Annex C^8^as follows: (1) basic demographic information, including familiarity with hiking , (2) sound source identification per sound type (sounds of technology, sounds of nature, sounds of human beings), (3) perceived affective quality of the present sound environment, (4) overall quality of the surrounding sound environment, (5) appropriateness of the surrounding sound environment to the present place. The Method A-type questionnaire was then expanded to capture more nuanced characterization of the sounds of nature, perceived overall visual quality of the present place, and participants’ experience in mountain sports to account for the possible effect of familiarity. The questionnaire was administered in Italian and English, referring to Aletta et al.^82^ for the translation of perceptual attributes. Questionnaire items are described in Table 8, while the complete questionnaire in Italian and English is provided in Supplementary Material.

**Table 8:** Questionnaire items in English and Italian.

Question code	Question		Question type
	English	Italian	
Q1	Please specify your age (in years)	Età	Open-ended question
Q2	How would you describe your gender?	Come descriveresti il tuo genere?	Categoric
Q3	Do you often (at least once a month) practice mountain sports? (e.g. hiking, outdoor climbing, skiing)	Pratichi spesso (almeno una volta al mese) attività sportiva in montagna? (ad es. sci, arrampicata in esterno, passeggiate)	Categoric
Q4	To what extent do you presently hear the following types of sound?	In questo momento, in che misura senti i seguenti tipi di suoni?	5-point Likert scale
Q4.1	Traffic noise (e.g. cars, buses, trains, airplanes)	Rumore da traffico proveniente dall’esterno (ad es. di auto, bus, treni, aerei)	
Q4.2	Other noise (e.g. sirens, construction, industry, loading of goods)	Altri tipi di rumori (ad es. sirene, cantieri, sorgenti, industriali, carico e scarico di merci)	
Q4.3	Sounds from human beings (e.g. conversation, laughter, children at play, footsteps)	Suoni prodotti da persone (ad es. conversazioni, risate, bambini che giocano, passi)	
Q4.4	Animal sounds (e.g. birds chirping, animals calling, insects buzzing)	Suoni di animali (ad es. cinguettio degli uccelli, canto di animali)	
Q4.5	Wind noise (e.g. rustling of trees)	Rumore del vento (ad es. fruscio degli alberi)	
Q4.6	Sound of flowing water (e.g. of a stream)	Suono dell’acqua (ad es. di un ruscello)	
Q5	For each of the 8 scales below, to what extent do you agree or disagree that the present surrounding sound environment is…	Per ciascuna delle 8 scale sottoostanti, in che misura sei d’accordo o meno sul fatto che l’ambiente sonoro che ) circonda sia:	5-point Likert scale
Q5.1	Pleasant	Piacevole, confortevole	
Q5.2	Chaotic	Caotico, confuso	
Q5.3	Vibrant	Vivace, stimolante	
Q5.4	Uneventful	Stabile, stazionario	
Q5.5	Calm	Calmo, tranquillo	
Q5.6	Annoying	Spiacevole, irritante	
Q5.7	Eventful	Dinamico, vario	
Q5.8	Monotonous	Monotono, noioso	
Q6	Overall, how would you describe the present surrounding sound environment?	Complessivamente, come descriveresti l’ambiente sonoro che ti circonda in questo momento?	5-point Likert scale
Q7	Overall, to what extent is the present surrounding sound environment appropriate to the present place?	Complessivamente, in quale misura l’ambiente sonoro che ti circonda in questo momento è appropriato al luogo in cui ti trovi?	5-point Likert scale
Q8	Overall, how would you describe the present surrounding visual environment?	Complessivamente, come descriveresti l’ambiente visivo che ti circonda in questo momento?	5-point Likert scale
Q9	Do you have any comment on this listening point? Write them here.	Hai altri commenti su questo punto di ascolto? Scrivili qui.	Open-ended question

A total of 443 questionnaires was submitted in paper form. Data was cleaned during the manual entry into a digital form. No full questionnaire was discarded but occasional missing data was observed, i.e. for certain questionnaire items, there are no more than 435 responses available.

### Participants

A total of 88 participants (Lago di Braies (*N* = 14), Val Venegia (*N* = 6), Passo Rolle (*N* = 18), Glen Lui (*N* = 25), Tre Cime di Lavaredo (*N* = 25)) have attended the five walks. The reported mean age was 35.6 years old, with youngest participant of the age 19 and the 77 being the eldest one, which makes for the age range of 58 years. Four participants didn’t report their age but were not excluded from the sample. 40 (45%) participants reported their gender as female, 45 (51%) as male and two (4%) preferred not answering the question. 59 (67%) participants reported that they often practice mountain sports such as hiking, outdoor climbing or skiing, while 29 (33%) participants reported that they do not practice those activities often. The majority of participants across the five walks were different, with a small possibility that a few attended multiple walks in Italy. This was not controlled for in the analysis due to the data anonymization process. The participants were recruited from the general public usually 1–2 months ahead of the soundwalk via public calls posted on social networks such as LinkedIn, Facebook and X. Data about the walking route, elevation, length and the duration were advertised in the call, allowing for a fitness self-assessment.

As the research involved human participants, the study design was reviewed and approved by the Ethics Committee at the Bartlett School of Environment, Energy and Resources, University College London (registered under Z6364106/2023/05/08 social research), while procedures in place at the Institutional Research Offices at EURAC Research and University of Trento were followed for questionnaire administration based on the principle of informed consent. The participants provided their informed consent in written form following the online distribution of the Participation Information Sheet prior to each soundwalk. Additionally, for all the soundwalks, a written informed consent for publication was provided by participants to show individual images in the research publications and social media, including online open access publications. All the methods were performed in accordance with the Declaration of Helsinki^83^.

### Audio recordings and environmental acoustic measurements

**Fig. 4:**
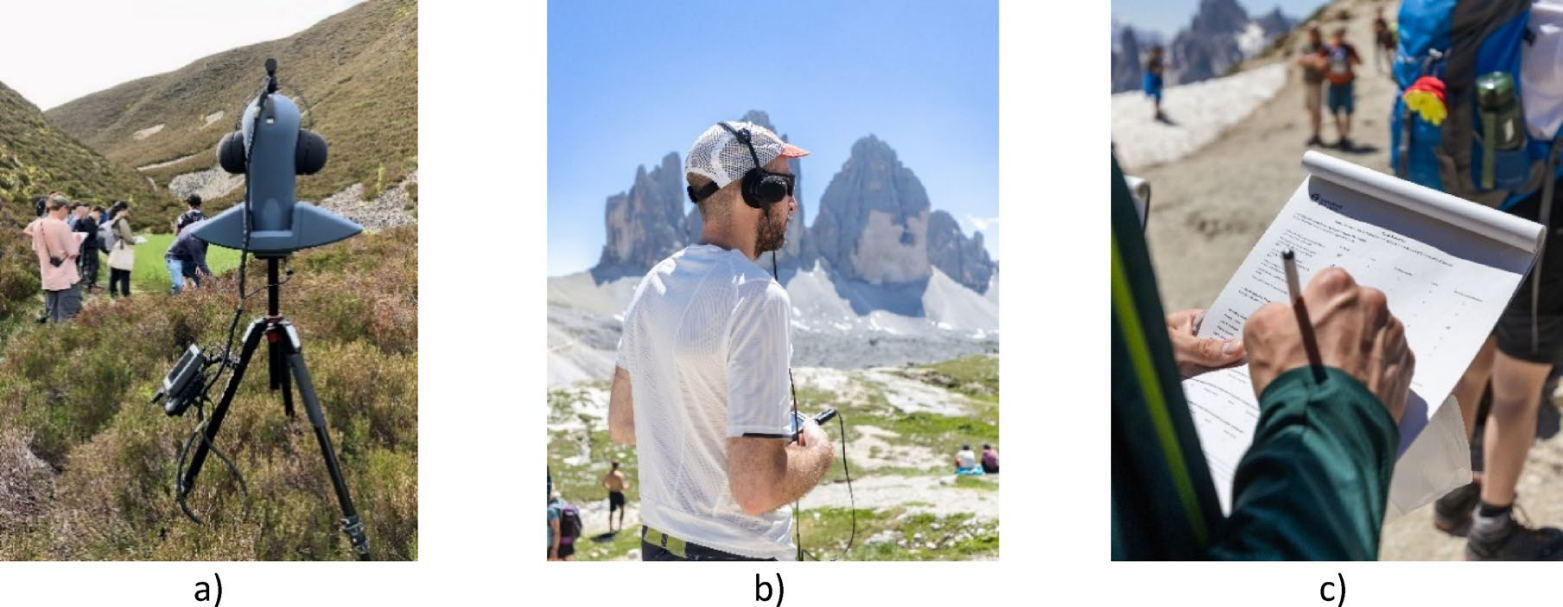
Data collection during soundwalks: (**a**) binaural recordings using a head and torso simulator in Glen Lui (data not used in this study), (**b**) recordings with a binaural headset at Tre Cime di Lavaredo, (**c**) completion of the questionnaire in paper format at Tre Cime di Lavaredo. Picture (**b**) and (**c**) credit: Mario Pedron.

All audio recordings and measurements were performed by an operator wearing the head-mounted binaural microphone kit (BHS II by HEAD acoustics) during the questionnaire, as shown in Fig. 4b). During some sessions a head and torso simulator was present as well, as shown in Figs. 4a and 6), but that data was not used in this manuscript as the priority was given to the head-mounted kit for consistency. The front end devices varied between the sessions (SQuadriga III and SQobold by HEAD acoustics), but all the systems were Class 1 compliant, with the BHS II – specific equalisation engaged and set to ID^84^and were calibrated following the same procedure using the 94 dB 1 kHz sine wave generator for all sessions.

### Procedure

**Fig. 5:**
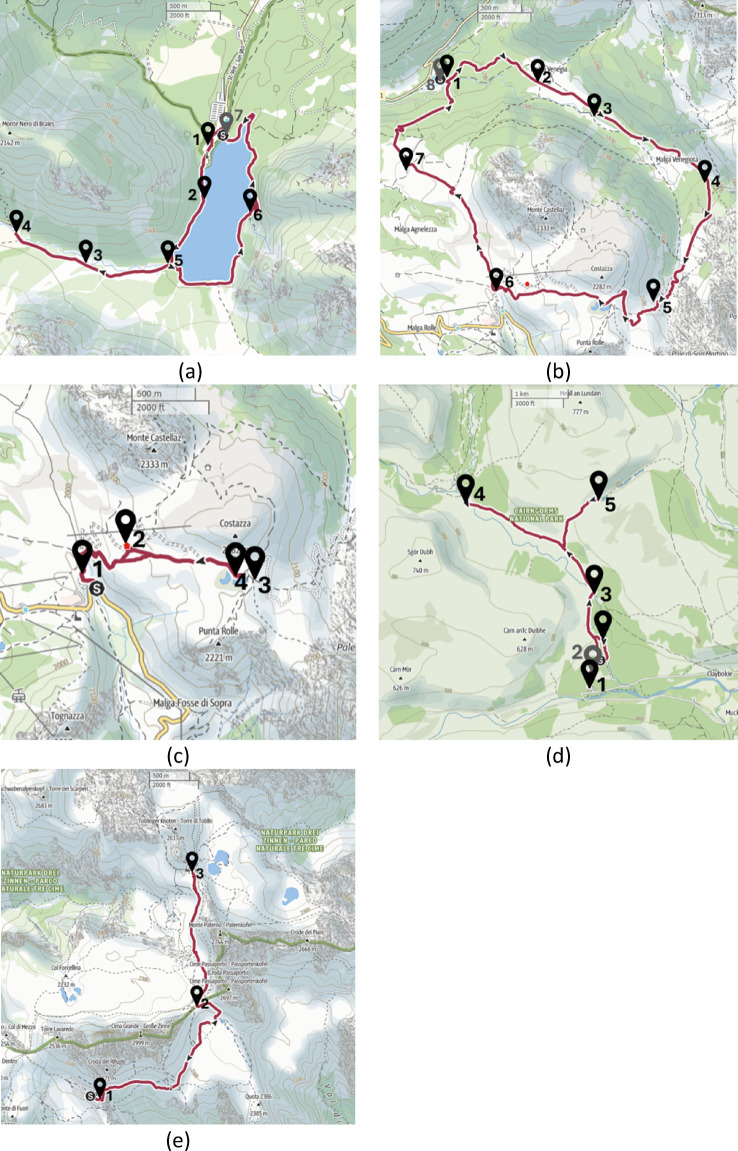
Overview of the soundwalks: (**a**) Lago di Braies (Italy), (**b**) Val Venegia (Italy), (**c**) Passo Rolle (Italy), (**d**) Glen Lui (Scotland, UK), (**e**) Tre Cime di Lavaredo (Italy). Numbers indicate listening stops. The scale is provided by the rulers. Dark green line represents the administrative borders of the protected area, dark red line represents the walking route, while the yellow line represents the main road. Source: OpenStreetMap through Outdooractive: https://www.outdooractive.com/en/^94^. All routes began and concluded at the same location.

Each of the five routes featured a number of listening stops. A total of 28 evaluation points (listening points) were recorded altogether (Lago di Braies (*N* = 7) – Fig. 5a, Val Venegia (*N* = 8) – Fig. 5b, Paso Rolle (*N* = 4) – Fig. 5c, Glen Lui (*N* = 6) – Fig. 5d, Tre Cime di Lavaredo (*N* = 3) – Fig. 5e). The exact locations of the listening stops, shown in Fig. 5, were recorded with the GPS tool integrated in the binaural measurement kit and added manually where the measurement device lost connection with the satellites.

All the five walking routes were selected so most of the stops are within the administrative borders of a protected natural area. It was expected that in a protected natural area where its management is focused on protection and tourism, visitors’ expectations of the overall sensory experience would be higher so the message about possible issues with environmental noise would be received as stronger. Moreover, one of the walks (Lago di Braies, Fig. 5a) was selected knowingly that there is a high chance of encountering crowds. Further considerations included accessibility by transport and the trail walkability for inexperienced hikers for the risk management purposes. All the routes were formed of the existing hiking trails, following recommendations from the official guides. The locations of the listening spots were decided ahead of the walks by observing two key criteria: (1) distance in relation to the whole walk for pragmatic reasons, (2) diversity of sonic experiences that were to be expected during the walk, based on scouting. The authors believe this kind of sampling is inevitable in studies that combine research with public engagement and the research focus is not jeopardized in any way, i.e. a completely random location sampling wouldn’t improve the level of quality at which the research questions are answered.

**Fig. 6:**
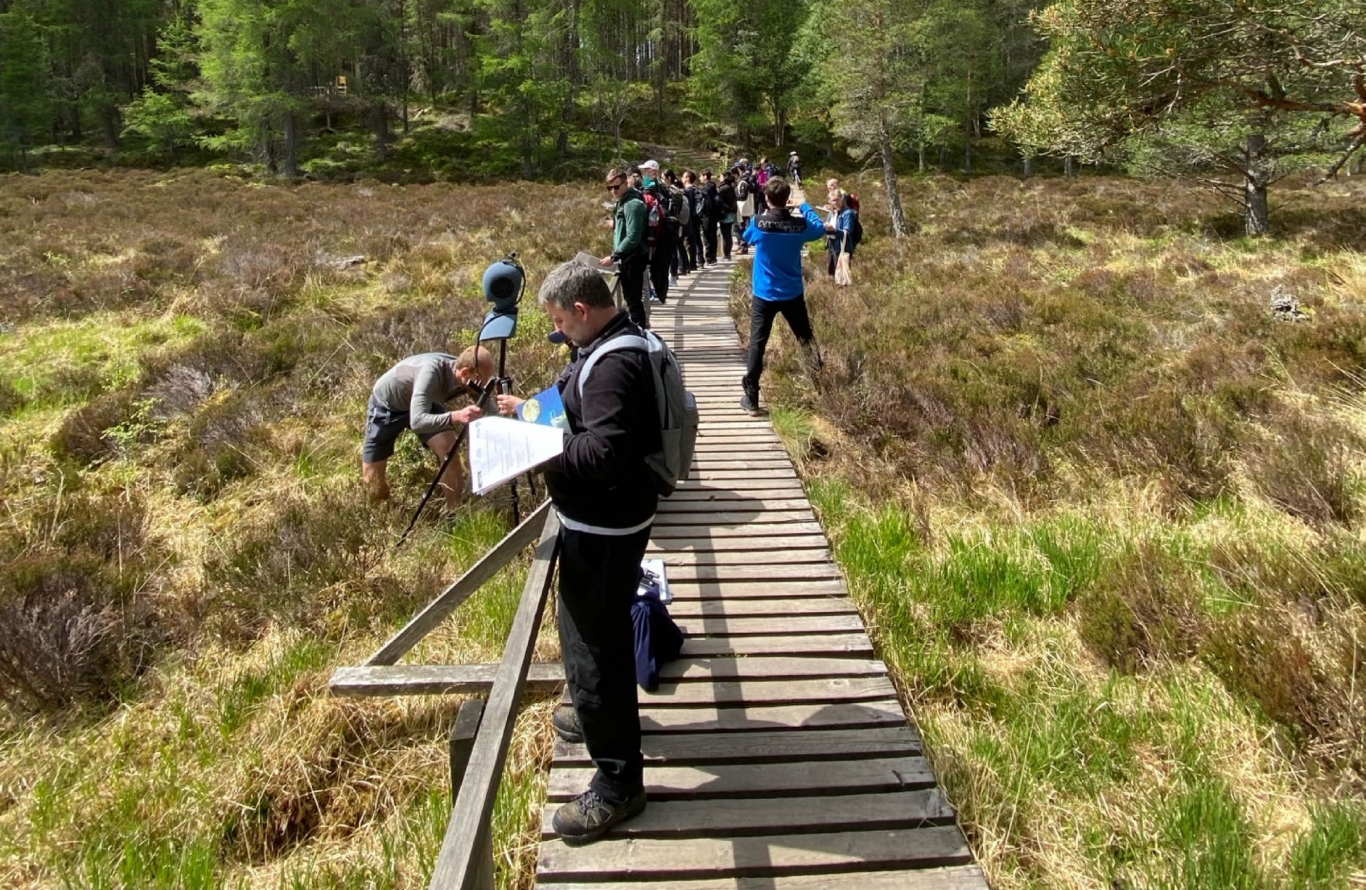
The operator with the head and torso simulator (data not used in this study – see Methods, Audio recordings and environmental acoustic measurements) and the participants in the same position, looking in the same direction, listening, then filling in the questionnaire, during the session in Glen Lui, Scotland. Picture credit: Mario Pedron.

Participants and researchers walked along the predefined route as a group. While walking, participants were free to talk and interact with each other as the typical visitors would do. To minimise the disturbance to other visitors and the environment, we have either sought advice from local guides or had them accompanying us on the walks, following their recommended behaviour patterns, i.e. walking in line, making space for other visitors, not damaging the undergrowth, giving particular attention to specific species. At each listening stop, researchers invited participants to choose a spot where they feel comfortable in relation to the walking path, topography, other participants and other visitors, and then face towards the same view as the researcher handling the binaural recording system or the head and torso simulator (Fig. 6), followed by listening in silence for a minute and filling in a questionnaire (Fig. 4c). Meanwhile, the researchers collected at least 3 min of calibrated binaural recordings before proceeding to the next listening point. This method aimed to ensure that the audio recorded by the operator corresponds to what participants heard while completing the questionnaire, accounting for certain small variability between the participants. During the expedition, team members also collected photos and video footage of the soundwalk for social media and outreach activities. However, care was taken not to disturb the listening moments, avoiding noise from cameras, operator movements, and drones.

### Data analysis

#### Data cleaning

A total of 28 audio recordings was made. A data cleaning protocol was performed where two researchers independently listened to each of the recordings and visually inspected spectrograms using software package ArtemiS SUITE 12.9^85^. Five recordings were discarded due to excessive wind noise and weren’t included in further acoustic analyses, as suggested by Lyons et al.^86^. During the same listening sessions, 1-minute excerpts were selected for the analysis, from the usually 3-minutes long recordings made on-site. This has proved to be a period that could be consistently applied to all the 23 recordings after discarding the parts affected by wind or handling noise.

#### Acoustic analysis

ArtemiS SUITE 12.9 software package^85^ was employed to calculate environmental acoustic metrics, following the recommendations from the ISO/TS 12913-2 and ISO/TS 12913-3^8,9^ as per Table 1.

#### Perceptual data

Following the recommendations from the Part 3 of the ISO/TS 12913^9^, the following formula has been applied to calculate coordinates of the perceptual outcomes of the eight attributes in the Q5 and enable interpretation within the two-dimensional perceptual space defined by the axes representing “ISO Pleasantness” and “ISO Eventfulness”:

$$ISO{\text{ }}\,Pleasantness = {\text{ }}{{[(e - u) + {\text{ cos45}}^\circ (ch - ca) + {\text{ cos45}}^\circ (v{\text{ }} - {\text{ }}m)]} \mathord{\left/ {\vphantom {{[(e - u) + {\text{ cos45}}^\circ (ch - ca) + {\text{ cos45}}^\circ (v{\text{ }} - {\text{ }}m)]} {\left( {{\text{4 }} + \!{\underline {\, {\sqrt {32} } \,}} } \right)}}} \right. \kern-\nulldelimiterspace} {\left( {{\text{4 }} + | \!{\underline {\, {\sqrt {32} } \,}} } \right)}}$$

$${\text{ISO}}\, Eventfulness = {\text{ }}{{[(e - u) + {\text{ cos45}}^\circ (ch - ca) + {\text{ cos45}}^\circ (v{\text{ }} - {\text{ }}m)]} \mathord{\left/ {\vphantom {{[(e - u) + {\text{ cos45}}^\circ (ch - ca) + {\text{ cos45}}^\circ (v{\text{ }} - {\text{ }}m)]} {\left( {{\text{4 }} + | \!{\underline {\, {\sqrt {32} } \,}} } \right)}}} \right. \kern-\nulldelimiterspace} {\left( {{\text{4 }} + | \!{\underline {\, {\sqrt {32} } \,}} } \right)}}$$

where *a* is annoying, ca. is calm, *ch* is chaotic, *e* is eventful, *m* is monotonous, *p* is pleasant; *u* is uneventful, *v* is vibrant.

### Statistical analysis

Ten Linear Mixed-Effects Models (LMM) were computed, as shown in Table 4, with the following aims: LMM1 to explore associations between soundscape perception and the perceived sound source dominance, perceived visual quality and soundscape, while accounting for individual age, gender, and habit of experiencing the mountains (regular vs. occasional visitor) (RQ1); LMM2 to LMM10 were designed as single parameter models and computed to test the ability of a set of nine acoustic and psychoacoustic metrics to predict soundscape perception. This approach was preferred to using multivariable models since the aim of the RQ2 was to provide findings easy-to-interpret and easy-to-implement in monitoring of PNAs at the sensor node, where simplicity and efficiency are critical, minimising storage and post processing issues^87^. Models are described in Table 9.

**Table 9:** Specification of model equations. Equal models were considered for both ISO Pleasantness (LMM_P) and ISO Eventfulness (LMM_E) scores.

Group fixed effect	*n*.	Model *R* code
Site perception	LMM1	~ Q1 + Q2 + Q3 + Q4.1 + Q4.2 + Q4.3 + Q4.4 + Q4.5 + Q4.6 + Q8 + (1|SiteID /EvaluationPointID) + (1|Participant_SiteID)
Measurements	LMM2	~ L _Aeq, T_ + (1|SiteID /EvaluationPointID) + (1|Participant_SiteID)
	LMM3	~ L_Ceq, T_-L_Aeq, T_ + (1|SiteID /EvaluationPointID) + (1|Participant_SiteID)
	LMM4	~ L _AF5,*T*_-L _AF95,*T*_ + (1|SiteID /EvaluationPointID) + (1|Participant_SiteID)
	LMM5	~ N _rmc_ + (1|SiteID /EvaluationPointID) + (1|Participant_SiteID)
	LMM6	~ N_5_/N_95_ + (1|SiteID /EvaluationPointID) + (1|Participant_SiteID)
	LMM7	~ T + (1|SiteID /EvaluationPointID) + (1|Participant_SiteID)
	LMM8	~ S + (1|SiteID /EvaluationPointID) + (1|Participant_SiteID)
	LMM9	~ R + (1|SiteID /EvaluationPointID) + (1|Participant_SiteID)
	LMM10	~ F + (1|SiteID /EvaluationPointID) + (1|Participant_SiteID)

The experimental activity employed two independent factors with different levels each: Site (five levels) as a between-subject factor, and Evaluation Point (between 3 and 7 levels depending on the Site) as a within-subject factor.

Considering the repeated-measure nature of the experimental design, the authors adopted Linear Mixed-Effects Models (LMM) using the statistical software R^88^ and the R packages *lme4*^89^, considering multiple LMMs for each dependent variable. The basic theory of the LMM is that subjects’ responses are the sum of fixed factors, which are the variables of interest controlled during the study, and random factors that can influence the covariance of the data.

Concerning the generation of the model, the independent variables used as fixed effects were survey scores and measured acoustic variables. Participants were treated as a random factor. A random intercept varying among Sites and Evaluation Points was included in each model concerning the nested random effects (i.e., Evaluation Points nested in Sites). In addition, a by-subject random intercept was added to estimate the variance in the outcomes related to the different individuals^90^. The specification of the general final model was as follows:

Dependent Variable ~ Independent Variable + (1|SiteID /EvaluationPointID) + (1|Participant_SiteID).

Ten models were created and tested for each dependent variable, i.e., ISO Pleasantness (LMM_P) and ISO Eventfulness (LMM_E) scores, thus resulting in a total of twenty computed LMMs.

LMMs were computed after verifying the assumption of normality and homogeneity of residual data distributions. Variance Inflation Factor (VIF) or Generalized VIF (GVIF), in case of categorical predictor, were computed to diagnose collinearity for each predictor.

Once the models were computed, it was of interest to carry out a comparison to select the one(s) with the highest predictive power given the data, especially within the (psycho)acoustic-based models (LMM_P and LMM_E 2 to 10) and between perceptual-based (LMM1_P and LMM1_E). The Akaike Information Criterion (AIC) was used to compare the quality of the hypothesised models. The model with the smallest AIC has the highest predictive power and a two unit difference on AICs (ΔAIC = 2) is usually considered a threshold for evidence of a difference in the models^91^. In addition, to compare the accuracy of the tested models and represent the proportion of the total variance explained by the fixed effects and by both fixed and random effects, the marginal (R^2^_m_) and conditional (R^2^_c_) coefficients of determination were generated for each model. Indexes were estimated using the function *r.squaredGLMM* from the *MuMIn package*^88,92^ to be interpreted using the recommended thresholds for a minimum (0.20), moderate (0.50), and strong (0.80) effect size^93^.

## Electronic supplementary material

Below is the link to the electronic supplementary material.

Supplementary Material 1.

## Data Availability

Research data is deposited at the Zenodo webpage https://zenodo.org/records/10253143.
