# Supplementary material for: Human sounds and associated tonality disrupting perceived soundscapes in protected natural areas
Source: Sci Rep. 2025 Aug 6;15:28759. doi: 10.1038/s41598-025-08524-y (PMC12328657; doi:10.1038/s41598-025-08524-y)
Supplement: Supplementary file 1 — Supplementary Material 1. [file 41598_2025_8524_MOESM1_ESM.pdf]

UNIVERSITÀ DI TRENTO

Punto di ascolto 1

Rimani in silenzio, ascolta l'ambiente sonoro che ti circonda e compila le seguenti domande.

— In questo momento, in che misura senti i seguenti tipi di suoni?
Si prega di barrare una sola risposta per ogni tipo di suono.

	Per niente	Poco	Moderatamente	Molto	Domina completamente
Rumore da traffico proveniente dall'esterno (ad es. di auto, bus, treni, aerei)	☐	☐	☐	☐	☐
Altri tipi di rumori (ad es. sirene, cantieri, sorgenti industriali, carico e scarico di merci)	☐	☐	☐	☐	☐
Suoni prodotti da persone (ad es. conversazioni, risate, bambini che giocano, passi)	☐	☐	☐	☐	☐
Suoni di animali (ad es. cinguettio degli uccelli, canto di animali)	☐	☐	☐	☐	☐
Rumore del vento (ad es. fruscio degli alberi)	☐	☐	☐	☐	☐
Suono dell'acqua (ad es. di un ruscello)	☐	☐	☐	☐	☐

— Per ciascuna delle 8 scale sottostanti, in che misura sei d'accordo o meno sul fatto che l'ambiente sonoro che ti circonda sia:
Si prega di barrare una sola risposta per ogni scala.

	Molto d'accordo	D'accordo	Né d'accordo, né in disaccordo	In disaccordo	Molto in disaccordo
**Piacevole, confortevole**	☐	☐	☐	☐	☐
**Caotico, confuso**	☐	☐	☐	☐	☐
**Vivace, stimolante**	☐	☐	☐	☐	☐
**Stabile, stazionario**	☐	☐	☐	☐	☐
**Calmo, tranquillo**	☐	☐	☐	☐	☐
**Spiacevole, irritante**	☐	☐	☐	☐	☐
**Dinamico, vario**	☐	☐	☐	☐	☐
**Monotono, noioso**	☐	☐	☐	☐	☐

— Complessivamente, come descriveresti l'ambiente sonoro che ti circonda in questo momento?

Ottimo	Buono	Né buono, né cattivo	Cattivo	Pessimo
☐	☐	☐	☐	☐

— Complessivamente, in quale misura l'ambiente sonoro che ti circonda in questo momento è appropriato al luogo in cui ti trovi?

Per niente	Poco	Moderatamente	Molto	Perfettamente
☐	☐	☐	☐	☐

— Complessivamente, come descriveresti l'ambiente visivo che ti circonda in questo momento?

Molto bello	Bello	Né bello, né brutto	Brutto	Molto brutto
☐	☐	☐	☐	☐

— Hai altri commenti su questo punto di ascolto? Scrivili qui.

____

____

____

Basic demographic data:

Please specify your age (in years): ________

How would you describe your gender?

- o Male (including transgender men)
- o Female (including transgender women)
- o Prefer to self-describe as ________ (non-binary, gender-fluid, agender, please specify)
- o Prefer not to say

Please indicate your ethnic origin:

- o Asian/Asian British
- o Black/Black British
- o Mixed
- o White/White British

Do you often (at least once a month) practice mountain sports?
(e.g., hiking, outdoor climbing, skiing)

- o Yes
- o No

LISTENING STOP 1

Please list sound sources you noticed in descending order starting with the most noticeable sound source.
Any number of listed sound sources is possible, but less than 8 is recommended.

____

____

____

— To what extent do you presently hear the following types of sounds?

	Not at all	A little	Moderately	A lot	Dominates completely
**Traffic noise (e.g., cars, buses, trains, airplanes)**	□	□	□	□	□
**Other noise (e.g., sirens, construction, industry, loading of goods)**	□	□	□	□	□
**Sounds from human beings (e.g., conversation, laughter, children at play, footsteps)**	□	□	□	□	□
**Animal sounds (e.g., birds chirping, animals calling, insects buzzing)**	□	□	□	□	□
**Wind noise (e.g., rustling of trees)**	□	□	□	□	□
**Sound of flowing water (e.g., of a stream)**	□	□	□	□	□

— For each of the 8 scales below, to what extent do you agree or disagree that the present surrounding sound environment is…

	Strongly agree	Agree	Neither agree, nor disagree	Disagree	Strongly disagree
**Pleasant**	□	□	□	□	□
**Chaotic**	□	□	□	□	□
**Vibrant**	□	□	□	□	□
**Uneveful**	□	□	□	□	□
**Calm**	□	□	□	□	□
**Annoying**	□	□	□	□	□
**Eventful**	□	□	□	□	□
**Monotonous**	□	□	□	□	□

— **Overall, how would you describe the present surrounding sound environment?**

Very good	Good	Neither good, nor bad	Bad	Very bad
☐	☐	☐	☐	☐

— **Overall, to what extent is the present surrounding sound environment appropriate to the present place?**

Not at all	Slightly	Moderately	Very	Perfectly
☐	☐	☐	☐	☐

— **Overall, how would you describe the present surrounding visual environment?**

Very good	Good	Neither good, nor bad	Bad	Very bad
☐	☐	☐	☐	☐

— **Do you have any comment on this listening point? Write them here.**

____

____

____

____

____

____

____

____

____
